# Freeze-dried amniotic membrane graft with a spongy layer in bilateral peripheral ulcerative keratitis: a case report

**DOI:** 10.1186/s12886-023-03129-3

**Published:** 2023-09-21

**Authors:** Clara Bertret, Loïc Leveziel, Juliette Knoeri, Cristina Georgeon, Céline Jamart, Nacim Bouheraoua, Vincent Borderie

**Affiliations:** 1 Sorbonne Université, GRC n°32, Transplantation et Thérapies Innovantes de la Cornée, AP- HP, Centre Hospitalier National d’Ophtalmologie des Quinze-Vingts, 28 rue de Charenton, Paris, F-75012 France; 2 Internal Medicine Department, AP-HP, Centre Hospitalier National d’Ophtalmologie des Quinze-Vingts, 28 rue de Charenton, Paris, F-75012 France

**Keywords:** Freeze-dried amniotic membrane, Ulcer, Inlay graft, PUK, Confocal microscopy

## Abstract

**Background:**

Peripheral ulcerative keratitis (PUK) is a group of inflammatory corneal ulcers with stromal thinning and peripheral localization. Amniotic membranes (AM) are used for their anti-inflammatory and healing properties. A freeze-drying process now allows maintaining the AM viable for a long time at room temperature without altering its physical, biological, and morphologic characteristics. The effectiveness of spongy freeze-dried amniotic membrane (FD-AM) graft with multimodal imaging in the management of severe corneal thinning PUK has not been reported.

**Case presentation:**

A 67-year-old Caribbean man histologically diagnosed with ulcerative colitis, was referred to our tertiary eye care center for a deep nasal juxtalimbal ulcer of the left eye. He was treated with topical steroids and antibiotics, methylprednisolone pulses, and oral prednisone. Due to continuous stromal thinning with 100 μm of residual corneal thickness, the decision was made to perform surgery. Conjunctival resection, inlay and overlay spongy FD-AM (Visio Amtrix® S, Tissue Bank of France, FR) were performed to preserve globe integrity. Despite tapering off oral steroids, PUK developed in the fellow eye on the 2 months follow-up. Treatment with human monoclonal antibody against tumor necrosis factor-alpha was initiated to control the active underlying inflammation. Six months following surgery, the ulcer was healed and corneal thickness in front of the former ulceration was measured at 525 μm on anterior segment-optical coherence tomography. Confocal microscopy confirmed the integration of the amniotic membrane between the corneal epithelium and the anterior stroma.

**Conclusion:**

Transplantation of FD-AM with a spongy layer was associated with restoration of normal corneal thickness in the PUK area. It seems to be a safe, effective, and easily accessible solution for the surgical management of PUK with impending perforation.

## Background

Peripheral ulcerative keratitis (PUK) is a group of inflammatory corneal ulcers with stromal thinning and peripheral localization. It begins with immune cellular infiltrates in the juxtalimbal cornea, followed by a crescent-shaped ulcer that appears parallel to the limbus. In 10 to 30% of cases, it is associated with scleritis, and in 40% of cases, both eyes are affected [1]. The risk of perforation increases in case of associated scleritis and decreases with the introduction of an immunosuppressant [2].

Medical management of PUK includes intense lubrication and antibiotics, along with topical and systemic steroids and immunosuppressants to control the underlying inflammatory process. In advanced stages of thinning, surgical procedures are necessary to preserve globe integrity.

Amniotic membranes (AM) are known for their anti-inflammatory, anti-bacterial, anti-angiogenic, healing, and analgesic properties, partly because they contain growth factors, cytokines, and metalloproteinases inhibitors. Their use in ophthalmology has been widely described in the surgical management of ocular surface conditions such as eye burns, corneal defects, infectious keratitis, pemphigoid, and Stevens-Johnson syndrome. Cryopreservation and freeze-drying are two different preservation methods. Sterilization followed by freeze-drying has the advantage of maintaining the AM viable for a long time at room temperature without altering its physical, biological, and morphologic characteristics [3].

We report a case of bilateral PUK with a high risk of perforation surgically treated with a FD-AM graft.

## Case report

**Fig. 1:**
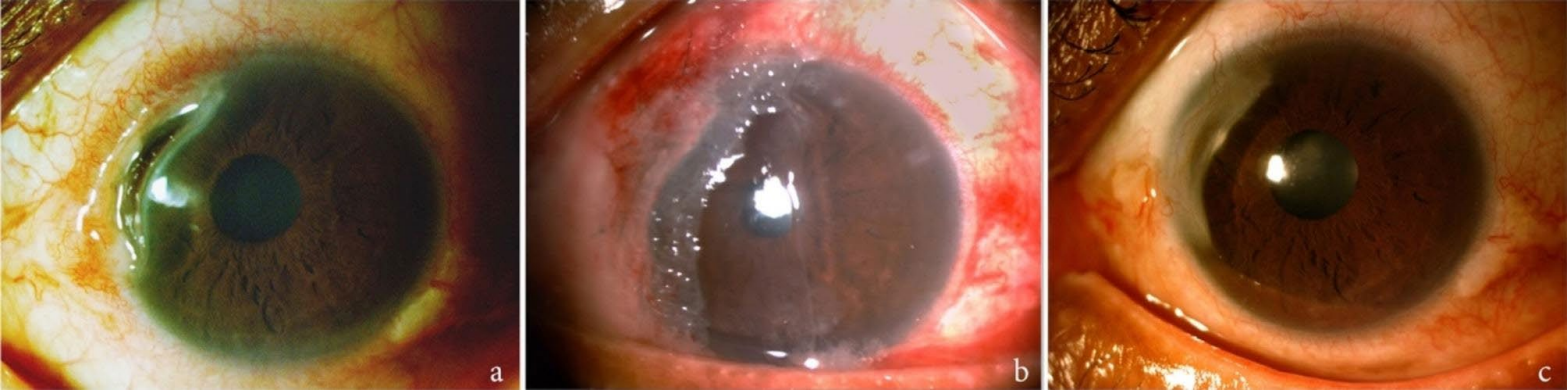
Slit-lamp photography of a 67-year-old man with peripheral ulcerative keratitis; left eye: (**1a**) Preoperative: peripheral juxtalimbal corneal ulcer with stromal lysis and scleritis, (**1b**) One day postoperative: spongy freeze-dried amniotic membrane in place after two-layers inlay graft followed by a single-layer overlay graft, (**1c**) Six months postoperative: freeze-dried amniotic membrane integrated

**Fig. 2:**
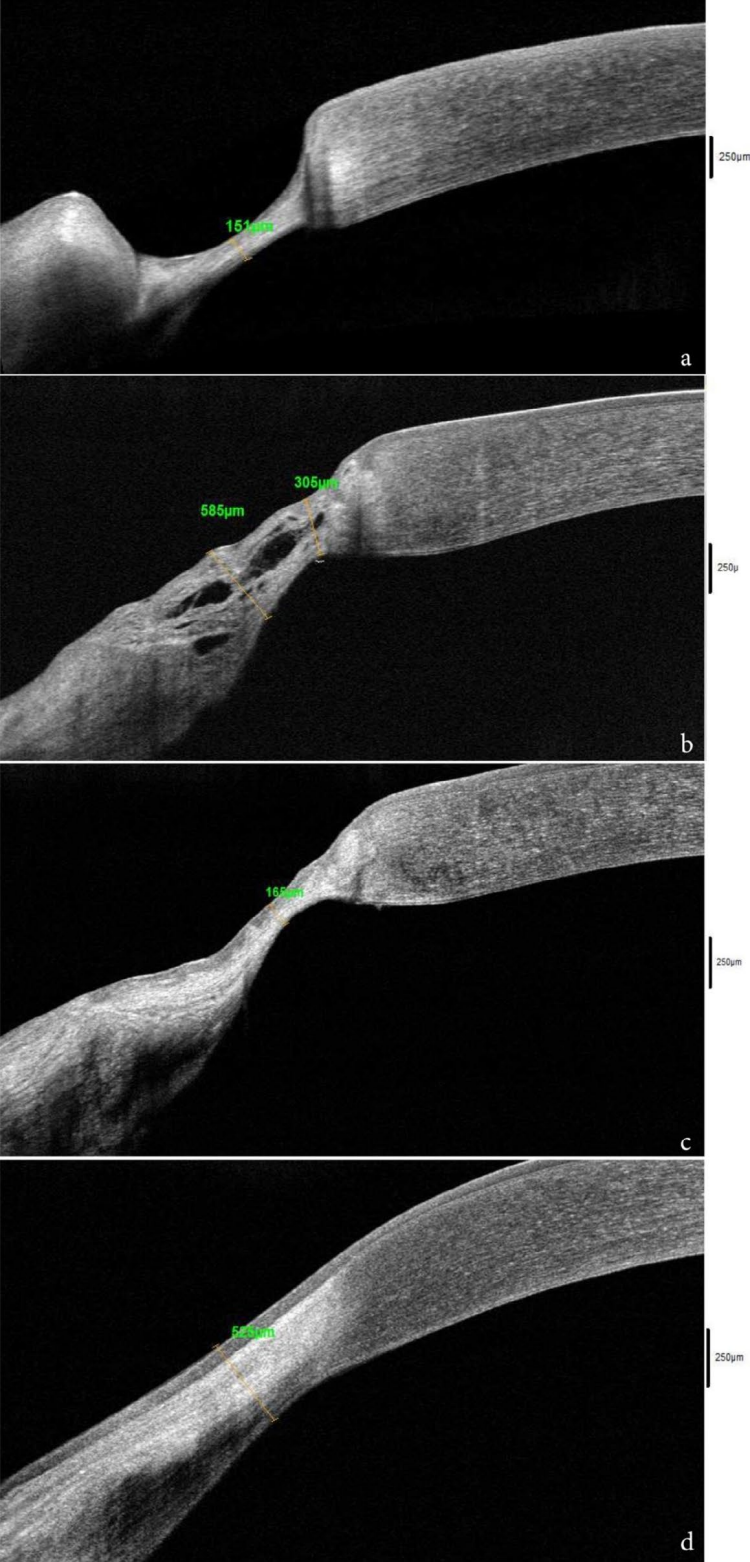
Anterior segment-OCT (RtVue-100®, Optovue®) scans of the thinnest zone of the corneal ulcer at various time points, left eye: (**2a**) Preoperative: peripheral stromal thinning with a minimal corneal thickness measured at 150 μm, (**2b**) Fifteen days postoperative: multilayer freeze-dried amniotic membranes integrated, (**2c**) One month postoperative: hyperreflective layers of amniotic membrane, (**2d**) Six months postoperative: epithelial and stromal thickening

A 67-year-old Caribbean man treated as conjunctivitis for 15 days was referred to our tertiary eye care center (Quinze-Vingts National Ophthalmology Hospital, Paris, France) for a deep nasal juxtalimbal ulceration of the left eye. His medical history revealed type 2 diabetes and asthma. Furthermore, following a routine medical check-up for colorectal cancer screening ten years earlier, he had been diagnosed with non-symptomatic ulcerative colitis that was confirmed histologically. On presentation, the patient complained of burning and tearing in his left eye. On examination, the best-corrected visual acuity was 20/20 in the right eye and 20/32 in the left eye. The intraocular pressure was 18 mmHg in the right eye and 16 mmHg in the left eye. Slit-lamp examination revealed a crescent-shaped left peripheral ulcer with mid-stromal corneal thinning, extending to the limbus from 8 to 10 o’clock with no lucid interval (Fig. 1a). A limbal inflammatory conjunctival bulge adjacent to the ulcer and scleritis were associated. Corneal sensitivity was preserved and no palpebral or blinking abnormalities were found. Slit-lamp examination of the right eye and dilated fundus exam were insignificant except for a right nasal pterygium. Anterior segment-Optical Coherence Tomography (AS-OCT) was performed and the minimal residual corneal thickness was measured at 150 μm (Fig. 2a).

The patient was diagnosed with PUK and treatment was given accordingly. Dexamethasone (1 mg) was injected sub-conjunctively. A topical steroid (dexamethasone 1 mg/ml) and a topical antibiotic (norfloxacin 3 mg/ml) were introduced 4 times a day. The patient was also treated with dorzolamide eye drops for ocular hypertension, non-preserved artificial tears, vitamin A ointment, and oral steroids (i.e., prednisone 0.5 mg/kg/day) followed by a deworming drug (ivermectin). Blood test investigations were performed by an internal medicine specialist before initiating systemic therapy.

The patient was seen five days later. He had failed to follow his oral steroids regimen and AS-OCT showed further thinning with 100 μm of residual corneal thickness. A microbiological corneal scraping was performed to rule out differential diagnosis. Because of worsening and impending perforation, the decision was made to perform emergency surgery to preserve globe integrity without waiting for the microbiological results. First, a 4 mm wide segment of perilimbal bulbar conjunctiva adjacent to the ulcer was resected. Then, an amniotic membrane graft was performed in two steps. Two layers of FD-AM with a spongy layer (Visio Amtrix® S, Tissue Bank of France, France) were successively sutured with interrupted 10 − 0 Nylon® to the deepithelialized thinned cornea as an inlay graft (epithelial side up). A third layer of spongy FD-AM was fixed to the conjunctiva using simple interrupted 8 − 0 Vicryl® sutures, as an overlay patch graft (chorion side up) (Fig. 1b). The patient received three intravenous pulses of methylprednisolone (1 g per day for 3 consecutive days) and was discharged on oral prednisone 40 mg per day.

Direct examination, microbiological culture, and polymerase chain reaction testing for herpes simplex virus, varicella-zoster virus, cytomegalovirus, and toxoplasmosis were all negative. Infectious diseases (tuberculosis, syphilis, Lyme, hepatitis C, and ascariasis) were also ruled out. No clinical nor biological arguments were found for connective tissue diseases or vasculitis such as rheumatoid arthritis, Wegener’s granulomatosis, periarteritis nodosa, Behçet’s disease, Horton’s disease, or sarcoidosis. A thoracoabdominal CT scan was performed and showed no deep adenomegaly and no identifiable parenchymal lesions. Although the patient had no digestive symptoms, the retained diagnosis was that of PUK in the context of ulcerative colitis.

**Fig. 3:**
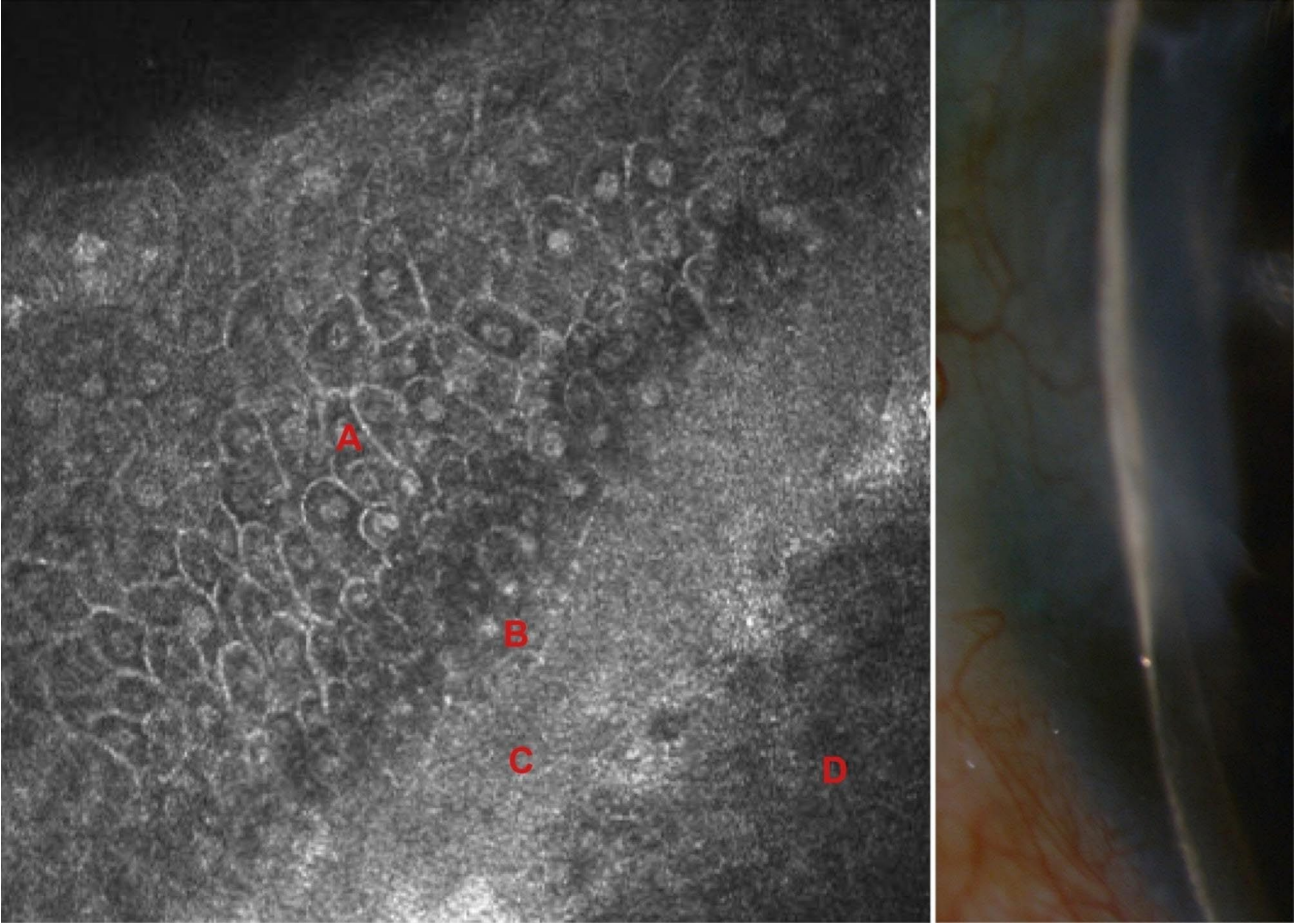
In vivo confocal microscopy (HRT3®, Heidelberg engineering®) and slit-lamp photography of the left eye six months after surgery: corneal epithelium (**A**), subepithelial nerve plexus (**B**), amniotic membrane (**C**), anterior stroma (**D**)

Two weeks after surgery, the juxtalimbal conjunctiva was no more inflammatory and the AM remained attached (Fig. 2b). Topical dexamethasone and oral steroids were progressively tapered. One month after surgery, the FD-AM graft was fully integrated without fluorescein staining. Corneal thickness in the area of the former ulcer was measured at 165 μm with AS-OCT (Fig. 2c). At two months, visual acuity improved to 20/25 in the left eye, and corneal thickness was measured at 340 μm with epithelial and stromal thickening opposite to the ulcer. Corneal inflammation of the left eye was under control, but examination of the fellow eye revealed a peripheral inferonasal ulcer with stromal thinning (431 μm) despite the patient still being on oral prednisone (15 mg/day). Dexamethasone eye drops were immediately initiated (4 times a day) and the right eye underwent the same surgical procedure: conjunctival resection, followed by a spongy FD-AM inlay graft and a spongy FD-AM patch graft. Three pulses of intravenous methylprednisolone (1 g per day for 3 consecutive days) were given and an immunosuppressive treatment with adalimumab (human monoclonal antibody against tumor necrosis factor-alpha) was introduced in the context of persistent inflammatory activity despite steroids. The postoperative follow-up was uneventful and systemic treatment was well tolerated. Six months after surgery of the left eye (Fig. 1c) and four months after the right eye, the corneal thickness was respectively 525 μm (Fig. 2d) and 540 μm in the areas of the former ulcers. In confocal microscopy, a hyperreflective layer corresponding to the spongy amniotic membrane was integrated between the newly formed corneal epithelium and the anterior stroma (Fig. 3). The epithelium covering the AM graft featured normal stratification with basal, wing, and superficial cell layers similar to the normal corneal epithelium. The AM was visualized as a homogeneous, acellular, hyperreflective layer as described [4].

## Discussion

The cornea benefits from an immunological privilege by being avascular and poor in immune cells in addition to other features including the anterior chamber associated immune deviation (ACAID). The corneal periphery is in contact with the vascular and lymphatic arcades and is, therefore, an exception to this privilege. Accumulation and deposition of immune complexes may activate the complement pathway and recruit inflammatory mediators [5]. In our patient, the release of collagenase and metalloproteinase led to severe keratolysis of the cornea.

Non-infectious corneal marginal ulcers are secondary to systemic disease in 50% of cases [6]. Wegener’s disease and rheumatoid arthritis are the two main vasculitis to be ruled out. Autoimmune ulcers are sterile but can be triggered by systemic infectious pathologies such as tuberculosis or syphilis. The diagnosis of an idiopathic Mooren’s ulcer cannot be established unless all other etiologies for PUK have been eliminated. Inflammatory bowel disease (IBD) is often associated with extraintestinal manifestations such as arthritis or erythema nodosum which evolve in parallel or independently of bowel symptoms. Ocular manifestations (episcleritis, scleritis, uveitis, PUK) have been reported to occur in less than 23% of ulcerative colitis cases, and may be associated with significant morbidity [7]. Corneal perforation from PUK is associated with high mortality rates, especially in the absence of immunosuppressive therapy [8].

In the early stages, PUK is usually managed with intense lubrication to promote healing, topical antibiotics to prevent secondary infections, topical steroids and ciclosporin to control the underlying condition. Steroids should be used in association with tears substitutes because they inhibit collagen synthesis and can delay scarring. In more advanced stages with impending perforation, a surgical approach is often needed to preserve globe integrity. Resection of the adjacent conjunctiva aims at reducing juxtalimbal source of the immune trigger and inflammatory cells, and AM grafting promotes rapid healing by releasing growth factors and reduces inflammation by sequestering inflammatory cells infiltrating the ocular surface [9]. The AM, with its anti-inflammatory properties, seems in theory more appropriate in PUK than cryanoacrylate glue. Howewer Yin J et al. show no difference in success in managing perforations with glue depending on the aetiology [10]. Only larger size of the perforation and single glue application were correlated with higher failure rate. Similarly, a tenon patch taken from the patient’s own inflamed eye seems less appropriate than an amniotic membrane. However, tenon grafts could be a solution in emergency situations [11]. These three different methods can also be successfully combined in current practice [12, 13]. Glue can also be used instead of sutures.

The AM is the inner layer of the fetal membrane and is composed of five adjacent layers: a single layer of epithelial cells, a basement membrane rich in collagen IV and V and laminin, a compact acellular layer, a fibroblast layer, and finally an outermost spongy layer. This spongy layer rich in collagen III, growth factors and proteoglycans provides additional thickness [14]. This FD-AM with spongy layer makes it more suitable for stromal reconstruction for deep corneal ulceration [15]. In their study, Nakamura et al. [3] showed that immunoreactivities were similar between FD-AM and cryopreserved AM and that neither lyophilization or gamma irradiation affects the biological or physical properties of the membrane. Also, no significant differences in total protein level and growth factor amount were found between the two types of AM [16]. Furthermore, FD-AMs can be stored at ambient temperature (between + 15 and + 25 °C) in the operating room and are easily accessible especially in emergency cases [17]. Therefore, the decision was made to use them for our patient with PUK. A multilayer graft was necessary because of the severe loss of stromal substance. The inlay graft is aimed at replacing the missing stroma and stimulating epithelial regrowth on top of the AM and the overlay patch at providing growth factors. Spongy FD-AM were able to successfully restore corneal thickness from 100 to 525 μm confirmed by AS-OCT and confocal microscopy.

Despite severe progressive thinning, the patient’s disease was adequately managed before corneal perforation occurs. If that had happened, a tectonic corneal graft may have been necessary which has to be systematically associated with immunosuppressive therapy because of the high risk of rejection [18]. Intravenous pulses of methylprednisolone allow for fast intense immunosuppression and are usually followed by oral prednisone (0.5 mg/kg/day) with gradual tapering. In nonresponding or severe cases, immunosuppressants such as cyclophosphamide, methotrexate, azathioprine, or biological agents are introduced. In our patient, worsening was seen despite steroid treatment, a human monoclonal antibody against tumor necrosis factor-alpha was introduced. Infliximab and Adalimumab supports remission of refractory ocular inflammation in patients with IBD. Adalimumab is given subcutaneously and the relatively low rate of side effects allows for better patient compliance [19–21].

## Conclusion

FD-AM graft with a spongy layer is a safe and effective alternative for the surgical management of PUK in combination with local and systemic immunosuppressive therapy. A close collaboration between the ophthalmologist and the internist is mandatory to prevent corneal perforation and decrease patient morbidity and risk of mortality.

## Data Availability

The datasets used and analysed during the current study available from the corresponding author on reasonable request.

## Abbreviations

AM: Amniotic membranes
FD-AM: freeze-dried amniotic membrane
PUK: Peripheral ulcerative keratitis
AS-OCT: Anterior segment-Optical Coherence Tomography
°C: Celsius degree
